# Early Life Stress and Brain Plasticity: From Alterations of Brain Morphology to Development of Psychopathology

**DOI:** 10.3390/neurosci3010008

**Published:** 2022-02-03

**Authors:** Fredrick Otieno Oginga, Thabo Magwai, Khanyiso Bright Shangase, Khethelo Richman Xulu, Thabisile Mpofana

**Affiliations:** 1 Department of Physiology, School of Laboratory Medicine and Medical Sciences, University of Kwa-Zulu Natal, Durban 4001, South Africa; 220111860@stu.ukzn.ac.za (T.M.); 210507028@stu.ukzn.ac.za (K.B.S.); xuluk2@ukzn.ac.za (K.R.X.); mpofana@ukzn.ac.za (T.M.); 2 Department of Clinical Medicine, School of Medical and Health Science, Kabarak University, Nakuru 20157, Kenya; 3 National Health Laboratory Service, Department of Chemical Pathology, University of Kwa-Zulu Natal, Durban 4001, South Africa

**Keywords:** early life stress, schizophrenia, psychopathology, anxiety, neuroplasticity, MD, brain development

## Abstract

Advances in our understanding of the genetics of mental disorders (MD) have contributed to a better understanding of their pathophysiology. Nonetheless, several questions and doubts remain. Recent research has focused on the role of the environment in developing mental disorders, and the advent of neuroscientific methodologies has opened up new avenues of inquiry. However, the mechanism by which childhood stress affects neurodevelopment via mechanisms, such as gene-environment interactions and epigenetic regulation leading to diseases in adulthood, is unclear. This paper aims to review the evidence on the role of early life stress and parental psychopathology in the pathophysiology and clinical expression of MD. **Methodology:** The study will conduct a comprehensive systematic review using medical search terms (MeSH). Electronic searches for published studies will be performed using the MEDLINE (PubMed), EMBASE, Scopus, PsychINFO, Web of Science, and Google Scholar databases. We will look for research on the neuroplasticity effects of early life stress on development and review articles that evaluate cognitive functions and the development of psychopathology and MD. Before identifying full-text articles, several studies will be filtered based on titles, abstracts, keywords, and synonyms. Publications to be included in the review will be assessed for quality and consistency before inclusion. Data will be extracted independently and duplicated by two authors from each eligible study to ensure consistency between reviews. All databases will be searched from inception until July 2021 and will be limited to human studies. The search will be limited only to publication in the English language and any publication that can be converted to English. **Discussion and Conclusions:** The findings of this review will meticulously articulate the effects of childhood adversity, such as ELS and parental psychopathology on cognitive development and neuroplasticity.

**Prospero Registration**: CRD42021278100

## 1. Background

Over the last decade, the long-term consequences of adverse childhood experiences have become a focus of a wide range of scientific inquiry. Early life stress (ELS), particularly childhood physical, sexual, and emotional abuse has been linked to poor general health outcomes in adulthood [1,2]. In a study of 44,066 people, those who had more than three ELS events had a four to twelvefold increase in addictive substance abuse, depression, and suicide [1]. Stress in early life has also been linked to personality disorders and the development of schizophrenia [3]. Childhood abuse, in particular, has been linked to higher levels of neuroticism later in life [4,5,6,7]. Despite the fact that epigenetic processes may be one way for early-life stress to become biologically embedded [8,9,10], altering how children respond physiologically and behaviorally to stress, for example, [11] discovered that early life stress facilitates changes in the cellular methylome at the glucocorticoid receptor gene (Nr3c1) and that these methylation differences persist [12] until adulthood, providing some of the most important evidence for early life epigenetic mediators [13,14,15,16], little research has been conducted into the neurophysiological underpinnings of the relationship between ELS and symptom development [17]. Given the plasticity of the central nervous system during childhood, it has been proposed that traumatic experiences during sensitive periods of brain development are likely to have adverse long-term effects on brain function and adaptation. Underscoring the centrality of stress to depression, many candidate biomarkers of depression, including smaller hippocampal volume [18], [19], disruptions in HPA-axis functioning [20,21] and elevated peripheral inflammation [22,23], are also linked with exposure to early adversity. However, there is a paucity of data on the possible physiological changes associated with adverse childhood experiences [24].

## 2. Review Questions

This study aims to illustrate the impact of early life stress on brain morphology and the development of psychopathology.

1. Investigate the neurobiological models of the effects of adversity on risk for mental disorder, including allostatic load, accelerated maturation, dimensional models, and sensitive period models on the development of psychopathology;
2. Evaluate the major mechanisms of neuroplasticity that unfold over the course of prenatal to adolescent development;
3. Investigate the Neuro-immune impacts of early-life stress on development and psychopathology;
4. Determine the relationship between early life adversity and the development of schizophrenia.

## 3. Method

### 3.1. Study Selection

Although many of the concepts discussed in this protocol are applicable to a wide range of mental disorders, including bipolar disorder, eating disorders, post-traumatic stress disorder (PTSD), and substance use disorders [25], we will use depression as an example to study how psychosocial adversity increases the risk of negative mental health outcomes by disrupting neurodevelopment [26,27] This systematic review will include the following criteria: (a) dichotomous or continuous measurement of ELS; (b) diagnostic assessment of depression using a clinical interview; (c) schizophrenia assessment before a mean age of 18 years; and (d) inclusion of a non-depressed control group.

### 3.2. The Domain of the Study

#### 3.2.1. Participant/Population

Inclusion criteria: Our inclusion criteria are as follows: All studies involving in vivo or in vitro human samples, brain and/or peripheral tissue. Participants with a history of adverse childhood experiences (ACE) according to validated measures (i.e., the Childhood Trauma Questionnaire [CTQ]) as well as a clinical diagnosis of MDD and/or MDD diagnosis according to validated scales (i.e., the Diagnostic and Statistical Manual (DSM)).

Exclusion criteria; our exclusion criteria include: All animal studies. Unpublished datasets, case studies, conference reports, non-refereed abstracts, observational studies, or multiple reports from the same dataset.

#### 3.2.2. Interventions/Interest

Inclusion criteria: Our inclusion criteria are as follows:

This study will show how early life adversity affects brain morphology and the development of psychopathology.

Exclusion criteria; our exclusion criteria include: Studies that do not show validated measures of assessment of childhood trauma/adversity. No assessment of schizophrenia using validated clinical measures.

### 3.3. Comparator(s)/Controls

The controls are the effects of early life stress and parental psychopathology on human cognition and neuroplasticity.

### 3.4. Types of Studies to Be Included

#### 3.4.1. Inclusion Criteria

To be included are all studies on the relationship between biomarker changes, early adversity in life, and the structural plasticity of neurons in the prefrontal cortices, amygdala, and hippocampus, and all studies involving human samples, brain and/or peripheral tissue, in vivo or in vitro.

#### 3.4.2. Exclusion Criteria

Our exclusion criteria include: All animal studies. Unpublished datasets, case studies, conference reports, non-refereed abstracts, observational studies, or multiple reports from the same dataset.

## 4. Main Outcomes

The main outcome of this systematic review is to determine the correlation between early adversity and parental psychopathology on the development of mental illness and the associated morphological changes in the brain associated with disease. We seek to amalgamate the differential changes in biomarkers, such as tumor necrosis factor-alpha (TNF-α), interleukin, C-reactive protein (CRP), structural and physiological plasticity of astrocytes, and neurons, through imaging studies in the prefrontal cortices, amygdala and hippocampus.

### 4.1. Measures of Effect

For each study, the major depressive disorder associated with early adversity, the differential levels of the tumor necrosis factor-alpha (TNF-α), interleukin-6 (IL-6), C-reactive protein (CRP), and the structural and physiological plasticity neurons in the prefrontal cortices, amygdala, and hippocampus will be collected.

### 4.2. Additional Outcome(s)

We will test whether demographic and methodological factors moderate the association between ELS and schizophrenia to obtain effect sizes. When available, the following demographic characteristics will be coded: (a) The sample’s average age at the time of depression assessment (in years); (b) the sample’s average age at the time of the ELS (in years). (c) Gender composition (proportion of female participants); (d) racial diversity (proportion Caucasian); and (e) continent on which the study was conducted. Furthermore, we will code for the following methodological characteristics: (a) sample size; (b) publication year; (c) research design (cross-sectional, longitudinal); (d) the source of the sample (student, clinic-referred, population-based, volunteer/community, or other/mixed); (e) depression diagnostic criteria used (e.g., DSM-III, DSM-IV); (f) presence of schizophrenia only vs. schizophrenia and other depressive disorders; (g) type of report used to assess early life stress (interview, questionnaire, or record review); (h) ELS assessment time frame (time-limited; whole-life); (i) ELS type (single measure or composite measure); (j) whether ELS interviewers were blinded to a schizophrenia diagnosis; (k) whether MDD interviewers were blinded to ELS; and (l) informant for schizophrenia and ELS (child, parent, or both). The original scales will be used to evaluate case-control and cohort studies, and a modified version 17 will be used to evaluate cross-sectional studies. Every effect size will be converted to an odd ratio (ORs). We will use the effect size values provided in the manuscript in publications where the study authors provided raw numbers.

### 4.3. Search Procedure

To identify the journal articles that will be included in this systematic review, we will employ several strategies based on the preferred reporting items for systematic review and meta-analysis protocols (PRISMA flowchart). First, we will conduct computer-based searches in Medline (PubMed), EMBASE, and PsycINFO for articles published in English between inception and July 2021, using the following keywords (or stems): (“Affective Disorder*” OR “Mood Disorder*” OR depress* OR MDD), AND (child OR childhood OR children OR infant OR adolescent OR adolescence) AND (child OR childhood OR children OR infant OR adolescent OR adolescence) AND (“early life stress” OR “early adversity” OR maltreatment OR “physical abuse” OR “sexual abuse” OR “emotional abuse” OR “psychological abuse” OR trauma OR neglect). Second, we will use forward and backwards searching to review bibliographies for additional studies. Third, we will send emails describing our systematic review and its inclusion criteria to professional membership LISTSERVs of research organizations, such as the American Psychological Association’s Society for a Science of Clinical Psychology, Association for Behavioral and Cognitive Therapies, and Society of Clinical Child and Adolescent Psychology.

### 4.4. Selection Process

The review will be based on qualitative and quantitative studies; there will be no study design, language, or time frame restrictions. Studies in languages other than English will be included if they can be sufficiently translated using Google Translate. Three authors, Fredrick Otieno Oginga (F.O.O.), Thabo Magwai (T.M.), and Khanyiso Bright Shangase (K.B.S.), will conduct the literature search. In cases where the three authors provide contradictory judgments, disagreements will be discussed, and the lead authors will decide.

### 4.5. Data Extraction

The authors, F.O.O., T.M., and K.B.S., will systematically screen all titles and abstracts for eligibility. Data will then be extracted using a standard data extraction form for (1) sample size, (2) gender distribution, (3) age of participants, (4) study design, (5) method of assessment for depressive and childhood trauma measures, and (6) relevant statistics (i.e., correlation values between childhood trauma and pro-inflammatory activity in patients with MDD). For studies where correlation values between childhood trauma and inflammatory measures were not directly reported, the corresponding author requested data, and the reviewers reported correlation measures.

### 4.6. Risk of Bias and Quality Assessment

The Downs and Black checklist will be used to assess the quality and biases of the studies that have been included [28]. The checklist is divided into four domains: bias reporting, external validity, internal validity, and selection bias. The checklist consists of 27 questions, with a possible score of 27. Downs and Black scores are classified as excellent (26–27), good (20–25), fair (15–19), and poor (0–14) [29]. Two independent authors will evaluate the quality of each included study (Fredrick Otieno Oginga, and Khanyiso Bright Shangase). In disagreements, the authors will discuss the studies in question, and the third author (Thabo Magwai) will make the final decision.

### 4.7. Statistical Analysis 

Statistical analysis will be conducted using STATA 17.0 software. ORs will be transformed using a logarithmic transformation. Pooled risk ratios (RR) will be calculated and reported with a 95% confidence interval. The heterogeneity will be quantified using the chi-square test and I2 metric. Depending on the level of heterogeneity, the pooled RR will be calculated with the fixed effects (FE) model or the random effects (RE) model. Dichotomous outcomes will be measured by using a risk ratio (RR) with a 95% confidence interval (CI). The Begg’s19 test and visual inspection of the corresponding funnel plots will be used to assess publication bias. To estimate heterogeneity of effect sizes, the standard Cochran’s Q Test will be used, which approximates a chi-square distribution with k−1 degrees of freedom, where k is the number of effect sizes, and the I2 provides the percentage of variability that is due to heterogeneity [30].

## 5. Discussion

Early life experiences have a significant impact on children’s neural, behavioral, and psychological development, with long-term consequences in a variety of domains [31,32]. Throughout the lifespan, experience shapes neural plasticity and, as a result, behavior and psychological processes. Infancy and early childhood have particularly high rates of synaptic regrowth and remodeling in the brain, and experiences can have long-term effects on development [33]. Prenatal and postnatal stressors are some of the forms of early stressors that could have developmental effects on the fetuses. Prenatal stress (PS) in humans is associated with an increased vulnerability to developing a variety of psychosocial problems in both childhood and adulthood. PS is linked to cognitive, behavioral, physical, and emotional problems in children, as well as autism and attention deficit hyperactivity disorder (ADHD). In adults, PS is linked to depression [34,35] and schizophrenia [36]. The pathophysiology of mental illness in people who have experienced early life adversity is directly related to neuroplasticity of the brain as a result of effects on neurons and inflammatory biomarkers. Although personalized medicine approaches that use biomarkers to guide patient treatment have had success in other fields of medicine, much progress remains to be made for those suffering from mental illness [37]. Molecular genetic advances have revealed links between immune genes, including those involved in the regulation of the inflammatory response, and the risk of mental illnesses, such as schizophrenia and bipolar disorders [38]. To better understand the pathophysiology of mental illness due early life adversity, it is important to study the changes in brain morphology and the deferential effects of stressors during early life in development of mental illness and schizophrenia. This study aims to illustrate the impact of early life stress on brain morphology and the development of psychopathology.

Review of team members and their organizational affiliations:

- Mr. Fredrick Otieno Oginga. The University of KwaZulu-Natal, College of Health Science, School of Laboratory Medicine and Medical Science, Department of Human Physiology.
- Mr. Thabo Magwai. The University of KwaZulu-Natal, College of Health Science, School of Laboratory Medicine and Medical Science, Department of Human Physiology.
- Mr. Khanyiso Bright Shangase. The University of KwaZulu-Natal, College of Health Science, School of Laboratory Medicine and Medical Science, Department of Human Physiology.
- Dr. Khethelo Richman Xulu. The University of KwaZulu-Natal, College of Health Science, School of Laboratory Medicine and Medical Science, Department of Human Physiology.
- Dr. Thabisile Mpofana. The University of KwaZulu-Natal, College of Health Science, School of Laboratory Medicine and Medical Science, Department of Human Physiology.

## Abbreviations

ACE	Adverse childhood experiences.
GRADE	Grading and recommendations assessment, development, and evaluation.
MeSH	Medical subjects headings.
PRISMA-P	Preferred reporting items for systematic review and meta-analysis protocols.
PROSPERO	Prospective register of systematic reviews.
MDD	Major depressive disorder or depression.
